# Neuroanatomical evidence and a mouse calcitonin gene–related peptide model in line with human functional magnetic resonance imaging data support the involvement of peptidergic Edinger–Westphal nucleus in migraine

**DOI:** 10.1097/j.pain.0000000000003294

**Published:** 2024-06-14

**Authors:** Ammar Al-Omari, Balázs Gaszner, Dóra Zelena, Kinga Gecse, Gergely Berta, Tünde Biró-Sütő, Péter Szocsics, Zsófia Maglóczky, Péter Gombás, Erika Pintér, Gabriella Juhász, Viktória Kormos

**Affiliations:** a Department of Pharmacology and Pharmacotherapy, Medical School, University of Pécs, Pécs, Hungary; b Department of Anatomy, Medical School and Research Group for Mood Disorders, Centre for Neuroscience, University of Pécs, Pécs, Hungary; c Institute of Physiology, Medical School, University of Pécs, Pécs, Hungary; d Department of Pharmacodynamics, Faculty of Pharmaceutical Sciences, Semmelweis University, Budapest, Hungary; e NAP3.0-SE Neuropsychopharmacology Research Group, Hungarian Brain Research Program, Semmelweis University, Budapest, Hungary; f Department of Medical Biology, Medical School, University of Pécs, Hungary; g Human Brain Research Laboratory, HUN-REN Institute of Experimental Medicine, Budapest, Hungary; h Szentágothai János Doctoral School of Neuroscience, Semmelweis University, Budapest, Hungary; i Department of Pathology, St. Borbála Hospital, Tatabánya, Hungary

**Keywords:** Migraine model, Centrally projecting Edinger–Westphal nucleus, Urocortin 1, Calcitonin gene–related peptide, Serotonin, Nucleus raphe dorsalis, Spinal trigeminal nucleus, fMRI

## Abstract

The urocortin 1 (UCN1)–expressing centrally projecting Edinger–Westphal (EWcp) nucleus is influenced by circadian rhythms, hormones, stress, and pain, all known migraine triggers. Our study investigated EWcp's potential involvement in migraine. Using RNAscope in situ hybridization and immunostaining, we examined the expression of calcitonin gene–related peptide (CGRP) receptor components in both mouse and human EWcp and dorsal raphe nucleus (DRN). Tracing study examined connection between EWcp and the spinal trigeminal nucleus (STN). The intraperitoneal CGRP injection model of migraine was applied and validated by light–dark box, and von Frey assays in mice, in situ hybridization combined with immunostaining, were used to assess the functional–morphological changes. The functional connectivity matrix of EW was examined using functional magnetic resonance imaging in control humans and interictal migraineurs. We proved the expression of CGRP receptor components in both murine and human DRN and EWcp. We identified a direct urocortinergic projection from EWcp to the STN. Photophobic behavior, periorbital hyperalgesia, increased c-fos gene–encoded protein immunoreactivity in the lateral periaqueductal gray matter and trigeminal ganglia, and phosphorylated c-AMP–responsive element binding protein in the STN supported the efficacy of CGRP-induced migraine-like state. Calcitonin gene–related peptide administration also increased c-fos gene–encoded protein expression, *Ucn1* mRNA, and peptide content in EWcp/UCN1 neurons while reducing serotonin and tryptophan hydroxylase-2 levels in the DRN. Targeted ablation of EWcp/UCN1 neurons induced hyperalgesia. A positive functional connectivity between EW and STN as well as DRN has been identified by functional magnetic resonance imaging. The presented data strongly suggest the regulatory role of EWcp/UCN1 neurons in migraine through the STN and DRN with high translational value.

## Abstract 2

Edinger–Westphal nucleus peptidergic neurons are activated in the calcitonin gene–related peptide model of migraine and their urocortinergic fibers may modulate the dorsal raphe and spinal trigeminal nuclei.

## 1. Introduction

Migraine is a neurovascular disorder^47,49^ that appears in the neurological practice as one of the most common complaints.^62^ Migraine attacks can last hours or days associated with headache, nausea, vomiting, photophobia, and phonophobia.^106^ The condition is attributed to activation and sensitization of the trigeminovascular system.^4^ Furthermore, a primary dysregulation of sensory processing may result in a constellation of neurological symptoms.^10^ The underlying mechanisms of migraine are not fully understood, and its therapy is not entirely resolved, highlighting the importance of further research.

The Edinger–Westphal (EW) nucleus includes a cholinergic preganglionic parasympathetic division that controls pupil constriction and lens accommodation, moreover, a peptidergic, centrally projecting division (centrally projecting EW [EWcp]).^57^ Interestingly, the latter is affected by stress,^54^ circadian rhythm,^35^ and ovarian hormones^18,19^ that are triggering factors of migraine attacks. The EWcp is the main source of central urocortin 1 (UCN1),^58,61,78,98,109^ a corticotropin-releasing hormone (CRH)–related neuropeptide that binds to both CRH receptors (CRH receptor 1 [CRH1R] and CRH receptor 2 [CRH2R]).^6,7,44,59^ Urocortin 1 has been implicated in control of food intake, energy homeostasis, stress response, mood level, and in response to acute pain.^32,53,57,84,109^ Centrally projecting EW projections include migraine-related centers, the spinal trigeminal (spinal trigeminal nucleus [STN]) and dorsal raphe nucleus (DRN),^109^ that express CRHRs,^14,15^ suggesting the possible ment of EWcp/UCN1 in migraine.

In the EWcp, several migraine-related neurotransmitters, neuromodulators, and receptors were found (eg, pituitary adenylate cyclase-activating polypeptide, pituitary adenylate cyclase-activating polypeptide type I receptor,^31,66,78^ neuronal nitric oxide synthase,^91^ substance P^65^), including calcitonin gene–related peptide (CGRP) fibers.^90^

Calcitonin gene–related peptide is a sensory neuropeptide with potent cerebral vasodilative activity,^13^ expressed abundantly in nociceptive trigeminal ganglion (TRG) neurons. Calcitonin gene–related peptide binds with equal affinity to calcitonin-like receptor (CLR) and calcitonin receptor (encoded by *Calcr* gene) when coexpressed with receptor activity-modifying protein 1 (RAMP1). The CLR/RAMP1 complex forms CGRP receptor and calcitonin receptor/RAMP1 complex forms amylin receptor1 (AMY1).^86^ Calcitonin gene–related peptide receptor component protein is not necessary for the receptor's activity, but it enhances the efficacy of CGRP.^20^

The CGRP, released by central nerve endings, stimulates the CGRP receptor on second-order neurons in the caudal division of STN, contributing to central sensitization.^8,46^ The trigeminal activation during migraine attacks elevates CGRP level.^39^ Human studies have reported migraine-like attacks following intravenous CGRP infusion.^40,60^ Serotonin (5-HT) receptor agonists (sumatriptan, dihydroergotamine) restore baseline CGRP level^38,85^ concurrent with pain relief.^50^

In spite that CGRP cannot pass the blood–brain barrier,^70^ intravenous CGRP administration can cause attacks in migraineurs,^2^ which can be reversed by CGRP receptor antagonist olcegepant infusion.^74^ Anti-CGRP and anti-CGRP receptor antibodies were effective in attack prevention,^16,94^ further suggesting a peripheral site of action.^3,28^

Other mouse studies demonstrated that CGRP may act both in the central nervous system (CNS) and periphery.^52,68,81^ Potential targets may be meningeal nociceptors leading to vasodilatation,^79^ the trigeminal system, cerebral blood vessels, and dura mater,^42^ but peripheral CGRP may alter the trigeminovascular microenvironment, leading to central sensitization.^25,29,45,46,68,81^

Here, we aimed to show neuroanatomical evidence that the EWcp/UCN1 may contribute to migraine. In a CGRP mouse model of migraine, we examined the EWcp and its migraine-related projection areas. Human functional magnetic resonance imaging (fMRI) studies were conducted to support the translational value of animal studies.

## 2. Materials and methods

### 2.1. Animals

Twelve-week-old male C57BL6/J mice were used and housed in a temperature and humidity controlled 12-hour light–dark cycle environment (lights on at 6 am) in standard polycarbonate cages (365 × 207 × 144 mm) in 4 to 6 mice per cage groups, at the animal facility of the Department of Pharmacology and Pharmacotherapy, University of Pécs. Mice were provided ad libitum with standard rodent chow and tap water. All procedures were approved by the Animal Welfare Committee at Pécs University, National Scientific Ethical Committee on Animal Experimentation in Hungary (BA02/2000-57/2022) in agreement with the directive of the European Communities Council in 1986, and with the Law of XXCIII, in 1998, on Animal Care and Use in Hungary.

### 2.2. Experimental design

#### 2.2.1. Neuroanatomical qualitative studies

Naive mice (n = 6) were used to examine the expression of AMY1 and CGRP receptor components in the EWcp, DRN, and STN as well as *Crhr1* and *Crhr2* mRNA in the STN.

Immunofluorescence targeting CLR and RNAscope in situ hybridization (ISH) targeting *Ramp1*, *Calcr*, and *Crcp* mRNA was combined with (1) UCN1 immunofluorescence in the EWcp to assess the colocalization with urocortinergic neurons in mice and in control human samples; (2) tryptophan hydroxylase-2 (TPH2) or 5-HT immunostaining in the DRN as a marker of the serotonergic neurons in mice, as TPH2 is the rate-limiting enzyme of the 5-HT synthesis; and (3) a neuronal marker (NeuN) immunofluorescence in the STN to visualize the dorsal horn neurons in mice.

To prove the connection between the urocortinergic EWcp neurons and STN, the EWcp of 6 naive mice was injected with an anterograde tracer adenoassociated virus serotype 8 (containing the green fluorescent protein [GFP] gene: AAV8 Syn enhanced GFP [EGFP]). Six animals received the injection of the retrograde tracer, cholera toxin subunit B (CTB), into their STN. To validate the anatomical localization of injections, CTB and GFP immunofluorescence was performed in the C1 segment of spinal cord and EWcp, respectively. For the retrograde tracing, UCN1 and CTB double immunofluorescence was performed on EWcp sections. For anterograde tracing, UCN1 and GFP double immunostaining was applied on C1 spinal cord sections.

Next, we investigated the expression of *Crhr1* and *Crhr2*, the receptor targets of UCN1, in the I-III laminae of the STN. For this purpose, RNAscope ISH for *Crhr1*, *Crhr2*, and *NeuN* was combined with UCN1 immunofluorescence in the STN. Our goal was to identify urocortinergic afferentation from EWcp to *Crhr1* and *Crhr2* positive neurons in the STN.

#### 2.2.2. Semiquantitative functional–morphological examination of the centrally projecting Edinger–Westphal in the calcitonin gene–related peptide model

Based on our neuroanatomical results, we aimed to prove the involvement of EWcp in a CGRP mouse model of migraine. Mice were handled for 2 weeks and assigned to saline-injected control (n = 11) and CGRP-treated (n = 15) groups. Upon intraperitoneal injection of 0.1 mg/kg CGRP or saline, light aversion was measured using light–dark box (LDB) test 30 minutes after the injection to assess photophobia associated with migraine-like state.

Another cohort of mice (n = 13) was used to assess periorbital hyperalgesia by the same model. Mice were subjected to the von Frey assay 30 minutes after the treatment.

The EWcp is known to be sensitive to various stressors.^34,36^ To exclude the changes in the EWcp caused by acute stress of LDB test, we used an independent cohort of mice for the functional–morphological studies. Two experimental groups were created: saline (n = 6) as a control group and CGRP-treated (n = 6) group. After 2 weeks of handling and habituation to intraperitoneal (i.p.) injections, mice were treated with 0.1 mg/kg of saline or CGRP (α-CGRP Mouse, Rat [CRB], Cat. No.: crb1000889), respectively. Mice were euthanized 4 hours after the treatment. Immunohistochemistry for the neuronal activity marker c-fos gene–encoded protein (FOS) was performed in the EWcp, lateral periaqueductal gray matter (lPAG), and laminae I to III of STN. In addition, we performed immunofluorescence for the alternative neuronal activity marker phosphorylated c-AMP–responsive element binding protein (P-CREB)^72^ in the STN. A whole mount FOS immunofluorescence was applied in the TRG to assess the neuronal activation in response to CGRP treatment. Double-label immunofluorescence for UCN1 and FOS was applied to prove the urocortinergic identity of activated EWcp neurons. *Ucn1* RNAscope ISH was combined with UCN1 immunofluorescence to assess UCN1 mRNA and peptide density in the EWcp. Serotonin (5-HT) and TPH2 double staining was performed to assess 5-HT and TPH2 density in the DRN.

#### 2.2.3. Targeted ablation of the centrally projecting Edinger–Westphal urocortinergic neurons

We performed stereotactic surgery to induce selective UCN1 neuron ablation using leptin-conjugated saporin in C57BL6/J mice (n = 13). Periorbital hyperalgesia in response to intraperitoneal injection of 0.1 mg/kg CGRP or saline was assessed before and 2 weeks after the surgery. Centrally projecting EW/UCN1 positive cells were counted to evaluate the saporin-induced urocortinergic neuronal loss using diaminobenzidine immunohistochemistry.

#### 2.2.4. Functional magnetic resonance imaging study

First, we performed the functional connectivity matrix analysis of EW in control humans, especially focused on the STN and DRN. Then, we compared it with interictal migraineurs' functional connectivity matrix. Finally, we examined the association between migraine frequency and the functional connectivity of EW.

### 2.3. Stereotaxic surgery in mice

Animals were anesthetized with i.p. ketamine-xylazine solution (16.6 mg/mL ketamine and 0.6 mg/mL xylazine-hydrochloride in 0.9% saline, 10 mL/kg) and fixed in a stereotaxic apparatus (David Kopf Instruments, Tujunga, CA).

For retrograde tracing, after a midline incision in the scalp and the nuchal skin, the muscles were detached from the occipital bone and reflected. Then, the posterior atlanto-occipital membrane was dissected and partially removed to visualize the spinal cord C1 segment that contains the STN. Under visual control using a surgical microscope, the glass capillary used for injection was moved 750 µm lateral to the posterior median sulcus in the midlevel of the space between the superior rim of the posterior arch of atlas and the inferior border of the occipital bone. Here, the tip of the capillary was perpendicularly introduced 100-µm deep into the laminae 2 to 3 of the STN. 2 × 20 nL of CTB (Cat. No.: #104; List Biological Laboratories, Campbell, CA) was injected in 2 steps with an automated injector. After 2 minutes, the needle was slowly removed. Finally, the muscles and the skin were sutured.

For anterograde tracing, AAV8 Syn EGFP (Addgene, Watertown MA, Cat. No.: 50465-AAV8; containing EGFP) was microinjected (2 × 10 nL) into EWcp. Here, we used the following stereotaxic coordinates from Bregma: posterior: −3.26; ventral: 3.35; lateral: 0. The injection was performed as described above for the STN, except that 1 minute after the injection, we lifted the needle by 0.5 mm, and upon additional 1 minute, it was slowly retracted from the brain, and the skin were sutured. Fourteen days after the surgery, both STN- and EWcp-injected animals were euthanized and transcardially perfused as described below.

### 2.4. Targeted toxin-induced lesion of centrally projecting Edinger–Westphal/urocortin 1 neurons

Saporin is a neurotoxin that enters neurons only if it is conjugated to a substance that is internalized by receptor-mediated endocytosis and irreversibly inhibits the cells' protein synthesis.^105^ Given that in the EWcp, only UCN1 immunoreactive neurons express leptin receptor, leptin-conjugated saporin injection provides a reliable tool to perform selective UCN1 neuron ablation.^95,107^ Fifty-nanolitre leptin-conjugated saporin (n = 13) (#KIT-47; ATS, Inc, Carlsbad, CA) was microinjected into the rostral (Bregma: posterior: −3.25; ventral: 3.75; lateral: 0) and caudal (Bregma: posterior: −3.75; ventral: 3.25; lateral: 0) part of the EWcp area. The injection was performed as described above for the anterograde tracer. Two weeks postinjection, mice were intraperitoneally injected with 0.1 mg/kg of either saline or CGRP, then subjected the von Frey assay. Mice were later perfused as described below. The neuronal loss was assessed and verified by UCN1 immunostaining. Two mice were excluded from this experiment because the histological assessment revealed that the injection path missed the EWcp area.

### 2.5. Light–dark box test

The light-aversive behavior was assessed using LDB test in the period between 30 and 60 minutes after 0.1 mg/kg i.p. injection of saline or CGRP.^68^ Mice were individually tested in the LDB device, consisting of 2 compartments connected by a small opening. The one chamber was brightly lit (1000 lux), whereas the other one was dark. Mice typically move in and out of the dark because of their curiosity to explore the novel environment; however, upon CGRP treatment, they prefer the dark compartment, in case of headache-like pain and photophobia.^68^ The time spent in the dark compartment was measured over 30 minutes to assess possible photophobia associated with migraine-like state.

### 2.6. Von Frey assay

Calibrated von Frey filaments were used to test periorbital hyperalgesia 30 minutes after 0.1 mg/kg i.p. injection of saline or CGRP. Each mouse was placed into a 10-cm-long restraining glass cylinder and allowed to poke out their heads and forepaws, but the restrainer prevented them from turning around.^30^ Mice were allowed to habituate for 5 minutes. The filament was applied to the periorbital region of the face (the midline of the forehead at the level of the eyes) in an ascending manner starting from the 0.04 g filament. Briefly, if an animal did not respond, increasing filament forces were applied until the 0.6-g filament was reached or until a response was observed.^5^ A positive response was defined as a sharp withdrawal of the head upon stimulation. Each filament was applied 5 times for 1 to 2 seconds with a 10-second interval. The periorbital withdrawal threshold was defined as the force at which the positive response occurred in 3 of 5 stimuli.^93^

### 2.7. Perfusion and tissue collection

Mice were euthanized by i.p. urethane (2.4 g/kg), then transcardially perfused with 20 mL of ice-cold 0.1 M phosphate-buffered saline (PBS) (pH 7.4) followed by 150 mL of 4% paraformaldehyde (PFA) solution in Millonig buffer (pH 7.4). Brain samples were dissected and postfixed for 72 hours in 4°C PFA solution. The brains were coronally sectioned using a Leica VT1000S vibratome (Leica Biosystems, Wetzlar, Germany). Four series of 30-μm sections were collected and stored in PBS containing sodium azide (0.01%) at 4°C, then for long-term storage in an antifreeze solution.

Four representative sections of the EWcp and lPAG (from Bregma −2.92 to −4.04), DRN (from Bregma −5.8 to −8.8 mm), and STN (spinal cord C1 segment) per animal were selected for each staining according to Paxinos.^77^ The trigeminal ganglion were collected and stored in a 4% PFA solution.

### 2.8. Human brain samples

Subjects (n = 3) studied in this project had no diagnosed neurological or psychiatric disorders or brain trauma, died from any non–brain-related cause and did not show any signs of brain neuropathologies (supplementary material Table 1, http://links.lww.com/PAIN/C72). After the removal of brains, perfusion through cannula placed into the internal carotid, and vertebral arteries was performed within a time window of 3 to 4 hours postmortem. Perfusion was commenced with 1.5 L of 0.33% heparin containing physiological saline for 30 minutes, then with 4 to 5 L of a Zamboni fixative solution containing 4% PFA and 0.2% picric acid in phosphate buffer (pH 7.4) over a duration of 1.5 to 2 hours.

The study received ethical approval from the Regional and Institutional Committee of Science and Research Ethics of the Scientific Council of Health (ETT TUKEB 15032/2019/EKU) and was conducted in adherence to the principles of the Declaration of Helsinki.

Tissue samples of the mesencephalic ventral periaqueductal gray matter were microdissected. The EWcp and DRN areas were identified according to the Allen human brain atlas^21^ and post-fixed in the Zamboni solution overnight.^92^ The brain samples were sectioned for 30-μm thickness using a Leica VT1000S vibratome (Leica Biosystems); then, the sections were collected and stored in PBS containing sodium azide (0.01%) at 4°C. For long-term storage at −20°C, they were transferred into antifreeze solution.

### 2.9. RNAscope in situ hybridization

The pretreatment procedure was optimized for 30 µm-thick PFA-fixed sections.^55^ Further steps (probe hybridization, signal amplification, and channel development) were performed according to RNAscope Multiplex Fluorescent Reagent Kit v2 user manual (ACD, Hayward, CA) to visualize the targets described in Table 1. Mouse (ACD; Cat. No.: 320881) and human triplex positive (ACD; Cat. No.: 320861) control probes and triplex negative (ACD; Cat. No.: 320871) control probes were tested on the samples. The triplex positive control probes gave well-detectable signal, whereas the negative control probes did not give any recognizable fluorescence in the preparations (images not shown).

**Table 1:** RNAscope in situ hybridization mRNA targets, respective probes and fluorophores.

Target	Probes	Fluorophores
*Crcp* mRNA	*Crcp*-C3 (Cat. No.: 810161-C3, ACD)	Cyanine3 1:750
*Ramp1* mRNA	*Ramp1-*C1 (Cat. No.: 532681, ACD)	Cyanine3 1:750
*Ctr* mRNA	*Calcr-*C2 (Cat. No.: 494071-C2, ACD)	Cyanine5 1:750
*Crh1r* mRNA	*Crh1r*-C1 (Cat. No.: 418011, ACD)	Cyanine3 1:750
*Crh2r* mRNA	*Crh2r*-C2 (Cat. No.: 413201-C2, ACD)	Cyanine3 1:750
*NeuN* mRNA	*Neun*-C3 (Cat. No.: 313311-C3, ACD)	Cyanine5 1:750
*Ucn1* mRNA	*Ucn1*-C1 (Cat. No.: 466261, ACD)	Fluorescein 1:3000

### 2.10. Immunofluorescence

After washes, sections were treated with 0.5% Triton X-100 (Sigma Chemical, Zwijndrecht, the Netherlands) in PBS for 30 minutes, and nonspecific binding sites were blocked with 2% normal donkey serum in PBS. Then, sections were incubated with the primary antibody (Table 2) for 24 hours. The secondary antibody treatment (Table 2) was applied for 3 hours at room temperature. Sections were counterstained with 4′,6-diamidino-2-phenylindole (ACD) and mounted on gelatin-coated glass slides, air-dried, and cover-slipped with glycerol-PBS (1:1).

**Table 2:** Antibodies used for immunostainings.

Target	Primary antibodies	Secondary antibodies
GFP	Chicken anti-GFP (Cat. No.: A10262, Life Technologies) 1:1000	Alexa 488-conjugated donkey antichicken (Cat. No.: 703-546-155, Jackson) 1:500
CTB	Goat anti-CTB (Cat. No.: #703, List Biological Laboratories) 1:5000	Alexa 488-conjugated donkey antigoat (Cat. No.: 705-545-003, Jackson) 1:500
UCN1	Rabbit anti-UCN1 (Cat. No.: ab283503, Abcam) 1:5000	Alexa 488-conjugated donkey antirabbit (Cat. No.: 711-545-152, Jackson) 1:500 or Cy3-conjugated donkey antirabbit (Cat. No.: 711-165-152, Jackson) 1:500
UCN1	Goat anti-UCN1 (Cat. No.: SC1825, Santa Cruz)1:250	Alexa 488-conjugated donkey antigoat (Cat. No.: 705-545-003, Jackson) 1:500
GFAP	Mouse anti-GFAP (Cat. No.: NCLLGFAP-GA5, Novocastra) 1:1000	Cy3-conjugated donkey antimouse (Cat. No.: 715-165-150, Jackson) 1:500
IBA1	Rabbit anti-IBA1 (Cat. No.: 019-19,741, Wako Ltd) 1:1000	Alexa 647-conjugated donkey antirabbit (Cat. No.: 711-605-152, Jackson) 1:500
P-CREB	Rabbit anti-P-CREB (Cat. No.: #9191, Cell Signaling) 1:500	Cy3-conjugated donkey antirabbit (Cat. No.: 711-165-152, Jackson) 1:500
CALCRL	Rabbit anti-CALCRL (Cat. No.: 703811, Invitrogen) 1:250	Cy3-conjugated donkey antirabbit (Cat. No.: 711-165-152, Jackson) 1:500
NeuN	Mouse anti-NeuN (Cat. No.: MAB377, Sigma-Aldrich) 1:1000	Cy3-conjugated donkey antimouse (Cat. No.: 715-165-150, Jackson) 1:500 or Alexa 488-conjugated donkey antimouse (Cat. No.: 715-545-150, Jackson) 1:500
5-HT	Goat anti-5HT (Cat. No.: ab66047, Abcam) 1:2000	Alexa 488-conjugated donkey antigoat (Cat. No.: 705-545-003, Jackson) 1:500
TPH2	Rabbit anti-TPH2 (Cat. No.: 348003, Synaptic Systems GmbH) 1:500	Alexa 647-conjugated donkey antirabbit (Cat. No.: 711-605-152, Jackson) 1:500 or Alexa 488-conjugated donkey antirabbit (Cat. No.: 711-545-152, Jackson) 1:500
FOS	Rabbit anti-cFOS (Cat. No.: 226 003, Synaptic Systems GmbH) 1:2000	Cy3-conjugated donkey antirabbit (Cat. No.: 711-165-152, Jackson) 1:500
FOS	Guinea pig anti-cFOS (Cat. No.: 226 005, Synaptic Systems GmbH) 1:1000	Alexa 488-conjugated donkey antiguinea pig (Cat. No.: 706-545-148, Jackson) 1:500

5-HT, serotonin; CTB, cholera toxin subunit B; FOS, c-fos gene–encoded protein; GFP, green fluorescent protein; P-CREB, phosphorylated c-AMP–responsive element binding protein; TPH2, tryptophan hydroxylase-2; UCN1, urocortin 1.

### 2.11. RNAscope in situ hybridization combined with immunofluorescence

After the RNAscope procedure (see above), slides were treated with the primary antibody (Table 2) for 24 hours. After washing, the slides were incubated with secondary antibody (Table 2) for 3 hours at room temperature in dark and then counterstained with 4′,6-diamidino-2-phenylindole (ACD), air-dried, and cover-slipped with glycerol-PBS (1:1).

### 2.12. Whole mount c-fos gene–encoded protein immunostaining

After 3 days of postfixation in 4% PFA, mouse TRGs were washed with PBS for 24 hours, incubated with 0.5% Triton X-100 for 6 hours (Sigma, Zwijndrecht, the Netherlands), and blocked using 2% normal donkey serum in PBS for 2 hours. Then, TRGs were incubated with FOS primary antibody (Table 2) for 48 hours. After washes (4 × 15 minutes) in PBS, samples were incubated with the Cy3-conjugated donkey antirabbit secondary antibody for 24 hours (Table 2), followed by 4 × 15 minutes PBS washes.

### 2.13. Immunohistochemistry with diaminobenzidine

The neuronal activity was assessed in EWcp, lPAG, and STN by FOS immunohistochemistry. The neuronal loss upon EWcp/UCN1 neuron ablation was quantified by UCN1 immunohistochemistry.

Sections were treated with 1% H_2_O_2_ (Sigma), permeabilized with 0.5% Triton X-100 (Sigma), and blocked with 2% normal goat serum in PBS. Subsequently, sections were incubated with rabbit anti-FOS or anti-UCN1 antibody (Table 2) overnight at room temperature. After washes, sections were incubated with biotinylated antirabbit gamma globulin for 1 hour (1:200 VECTASTAIN Elite ABC-HRP Kit; Peroxidase Rabbit IgG Vector Laboratories Inc. Newark, CA, *Cat. No.: PK-6101*, produced in goat). Then, sections were incubated in avidin–biotin complex solution for 1 hour. After washes, the labeling was developed with 0.05% diaminobenzidine (DAB) in Tris buffer with 0.06% H_2_O_2_ (Sigma). The reaction was controlled under a stereomicroscope and stopped with Tris buffer. After washes, sections were mounted on gelatin-coated slides, air-dried, treated with xylene (Merck, Leicester, United Kingdom), and cover-slipped with Depex mounting medium (Merck).

### 2.14. Double-label immunofluorescence for c-fos gene–encoded protein and urocortin 1

Urocortin 1 and FOS immunofluorescence was applied to prove the urocortinergic identity of activated EWcp neurons. Sections were washed with PBS, incubated with 0.5% Triton X-100 (Sigma) in PBS for 30 minutes and treated with 2% normal donkey serum in PBS. Anti-UCN1 rabbit antibody (Table 2) and anti-cFOS guinea pig (Table 2) antibody cocktail was applied overnight, at room temperature. After washes, sections were incubated with Cy3-conjugated donkey antirabbit and Alexa 488-conjugated donkey antiguinea pig antisera (Table 2) for 3 hours. After washes, sections were mounted on gelatin-coated glass slides, air-dried, and cover-slipped with glycerol-PBS (1:1).

### 2.15. Microscopy, digital imaging, and morphometry

The diaminobenzidine-labeled sections were studied and digitalized using a Nikon Microphot FXA microscope with a Spot RT camera (Nikon, Tokyo, Japan). The number of FOS-positive nuclei was determined by manual cell counting on the whole cross-section surface area of the EWcp, lPAG and STN on 4 sections per animal. The average of these 4 values represented the FOS activation of one mouse in the given brain area. For the targeted ablation, the number of UCN1-positive neurons was determined by manual cell counting on the whole cross-section surface area of the EWcp on all sections per animal. The sum of these values represented the number of UCN1 neurons of one mouse in the EWcp.

Fluorescent-labeled sections were digitalized by an Olympus FluoView 1000 confocal microscope (Olympus, Europa, Hamburg, Germany) by sequential scanning in analogue mode. We used 3.5-µm optical thickness, 1024 × 1024-pixel resolution for scanning. The excitation and emission spectra for the respective fluorophores were selected using built-in settings of the FluoView software (FV10-ASW; Version 0102, Olympus Europa). 4′,6-Diamidino-2-phenylindole was excited at 405 nm, Cy3 at 550 nm, Cy5 at 650 nm, Fluorescein and Alexa 488 at 488 nm. Sections were scanned for the respective wavelengths at 4 channels. Digital images of the individual channels, depicting the same area, were automatically superimposed and merged.

The UCN1, TPH2, and 5-HT immunofluorescence and the confluent or cluster-like *Ucn1* RNAscope signal was measured by Image J software (version 1.42.; NIH, Bethesda, MD) in 5 to 20 cell bodies using 4 nonedited images of the corresponding channel. The region of interest was manually determined at cytoplasmic areas of neurons. The signal density was corrected for the background signal. The average of the specific signal density (SSD) of 5 to 20 neurons was determined in 4 sections per animal. The average of these 4 values represented the SSD value of one mouse. The SSD was expressed in arbitrary units.

In human samples, autofluorescence caused by lipofuscin accumulation disturbed the imaging. Because the lipofuscin accumulation is characteristic for the cytoplasm, but not for the karyoplasm, we show high-magnification images including the cross-section profiles of neuronal nuclei. With this strategy, nuclear mRNA signal dots get well-distinguishable from cytoplasmic lipofuscin-related autofluorescence that appears in all channels.

### 2.16. Statistical analysis

Data were expressed as mean ± SEM for each experimental group. Data sets were tested for normality by Shapiro–Wilk test^87^ and evaluated using Student *t* test (Table 3). Data sets obtained in the experiment of targeted UCN1 neuron ablation were tested for homogeneity of variance and for normal distribution. Repeated-measures analysis of variance test was conducted to assess the effect of CGRP treatment before and after EWcp/UCN1 ablation (as within subject factor) on the pain threshold values, obtained in the von Frey test. The post hoc comparisons were performed by Tukey post hoc test. Analyses were performed with the software Statistica 8.0 (StatSoft, Tulsa, OK) (α = 5%).

**Table 3:** Summary of statistical results by Student *t* test analyses.

Measured parameters	*P*
Light dark box test	**0.02**
Von Frey test	**8.25 × 10** ^**−**^ **^13^**
FOS-positive neurons in the TRG	**0.0004**
FOS-positive neurons in the STN	0.18
FOS-positive neurons in the lPAG	**0.002**
P-CREB–positive neurons in the STN	**0.003**
FOS positive neurons in the EWcp	**0.03**
Urocortinergic FOS immunopositive neurons in the EWcp	0.44
*Ucn1* mRNA SSD in the EWcp	**0.0004**
UCN1 peptide SSD in the EWcp	**0.0007**
5-HT peptide SSD in the DRN	**0.03**
TPH2 peptide SSD in the DRN	**0.01**
UCN1 immunoreactive neurons in the EWcp upon LS-targeted ablation	**0.0002**

5-HT, serotonin; DRN, dorsal raphe nucleus; EWcp, centrally projecting Edinger–Westphal nucleus; FOS, c-fos gene–encoded protein; lPAG, lateral periaqueductal gray matter; LS, leptin-saporin; P-CREB, phosphorylated c-AMP-responsive element binding protein; SSD, specific signal density; STN, spinal trigeminal nucleus; TPH2, tryptophan hydroxylase-2; TRG, trigeminal ganglion; UCN1, urocortin 1.

Significant values are highlighted in bold.

### 2.17. Functional magnetic resonance imaging

#### 2.17.1. Participants

In the functional connectivity analysis, 35 migraine patients according to International Classification of Headache Disorders third edition^75^ diagnosis of episodic migraine without aura (27 women and 8 men, mean age ± SD = 25.24 ± 4.35 years) and 41 healthy controls volunteers (25 women and 16 men, mean age ± SD = 26.00 ± 4.59 years) were included. All participants were screened by headache specialists, free from any serious medical, neurological (except migraine without aura) or psychiatric disorders, have not taken any daily medications (except oral contraceptives), and they were right handed. The fMRI experiment was carried out in the interictal period of migraine patients, and they were free from migraine attack 48 hours before and 24 hours after the fMRI session. Their average migraine frequency was 3.36 ± 3.11 attacks per month.

The human fMRI study protocol was approved by the Scientific and Research Ethics Committee of the Medical Research Council (Hungary) (23609-1/2011-EKU [747/PI/11], 23421-1/2015/EKU [0178/15]). The entire study was conducted according to the Declaration of Helsinki and with the written informed consent of each participant.

#### 2.17.2. Functional magnetic imaging acquisition and seed region definition

The fMRI session started with the acquisition of a high-resolution structural data using T1-weighted 3D turbo field echo sequence and 1 × 1 × 1 mm^3^ resolution in a 3 T MRI scanner (Achieva 3 T; Philips Medical System, Eindhoven, the Netherlands). The resting-state fMRI session lasted 6 minutes when the participants were instructed to close their eyes but remain awake. The imaging data set acquisition parameters of T2*-weighted echo-planar imaging pulse sequence were the following: repetition time = 2.500 ms, echo time = 30 ms, field of view = 240 × 240 mm^2^; with 3 × 3 × 3 mm^3^ resolution. The state-of-the-art preprocessing pipeline was applied on raw data based on previous analysis of the research group.^37^

After the preprocessing steps, seed-to-voxel analysis was conducted with the EW (Montreal Neurological Institute [MNI] coordinates: x = 0; y = −23; z = −7, radius: 2 mm). The seed definition was carried out based on literature data^108^ and confirmed by the visual check of expert scientists. The spherical mask of the region was created using fslmaths command of FSL (Functional MRI of the Brain Software Library), whereas for the extraction of time-series data and subsequent computations of voxel-wise connectivity analysis, the NiBabel and NumPy modules were used. The seed-based connectivity map for each participant, established through voxel-wise Pearson correlation with the averaged seed region data, underwent transformation into Z-scores through Fisher transformation. These Z-score maps for each individual were subsequently employed in both within-group and between-group comparisons using the Statistical Parametric Mapping software package (Wellcome Department of Imaging Neuroscience, Institute of Neurology, London, United Kingdom). Given the fact that the EW is lying within the periaqueductal grey matter (PAG), we examined the extent to which we could differentiate its connectivity from those of the PAG using previously applied PAG seeds.^39^ Both similarities and differences were revealed in its connections, which are detailed in the supplementary material (Figure S1 and Table S2, http://links.lww.com/PAIN/C72).

#### 2.17.3. Functional connectivity analysis

The functional connectivity of EW was determined in whole-group analysis using one sample *t* test. After, 2 sample *t* test was conducted to compare the EW connectivity between migraine and control groups. A correlation analysis was used to investigate the relationship between migraine frequency and EW connectivity in migraine patients. To confirm the connection identified in animal experiments between EW with STN (MNI coordinates: left STN x = −6, y = −42, z = −48; right STN x = 6, y = −42, z = −48; radius: 4 mm) and DRN (from Harvard Ascending Arousal Network Atlas),^26^ region-of-interest analysis was conducted with these 3 regions.

All analysis was corrected for sex, age, and motion by adding them as covariates of no interest. An initial threshold of *P* < 0.001 uncorrected for multiple comparison and at least 20 contiguous voxels was used in the whole-brain analyses. All reported results survived family-wise error correction at a cluster-level threshold of p_FWE_ < 0.05. In the ROI analysis, the initial threshold of *P* < 0.001 uncorrected for multiple comparison was used, and the reported results survived family-wise error correction at peak-level threshold of p_FWE_ < 0.05.

For data visualization, statistical maps of significant clusters were used as overlay on MNI 152 template brain in MRIcroGL.^82^

## 3. Results

### 3.1. Neuroanatomical qualitative studies

#### 3.1.1. Amylin receptor1 and calcitonin gene–related peptide receptor components are expressed in the centrally projecting Edinger–Westphal, dorsal raphe nucleus and spinal trigeminal nucleus

**Figure 1.**
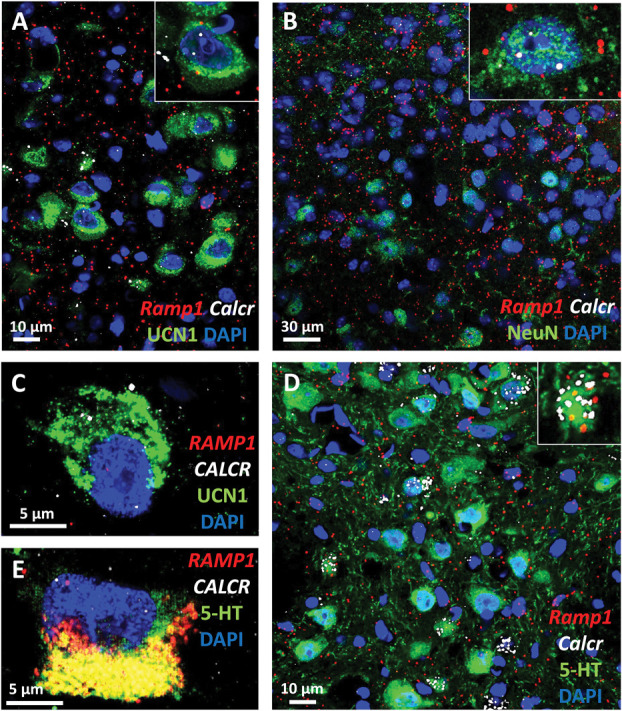
Calcitonin receptor (*Calcr*) and receptor activity–modifying protein 1 (*Ramp1*) mRNA expression. (A) Representative fluorescence images showing the *Calcr* (white) and *Ramp1* (red) mRNA transcripts coexpressed with urocortin 1 peptide (UCN1, green) in the mouse centrally projecting Edinger–Westphal (EWcp) nucleus. (B) Neurons (neuronal marker NeuN, green) of the mouse spinal trigeminal nucleus express both of *Calcr* (white) and *Ramp1* (red) mRNA. (C) Representative fluorescence images showing the *CALCR* (white) and *RAMP1* (red) mRNA transcripts coexpressed with UCN1 (green) in the human EWcp. (D) In the mouse dorsal raphe nucleus (DRN), the serotonin (5-HT, green) immunoreactive cells also contained *Calcr* (white) and *Ramp1* (red) mRNA transcripts. (E) In the human DRN, the 5-HT (green) immunoreactive cells also contained *CALCR* (white) and *RAMP1* (red) mRNA transcripts. The yellow cytoplasmic area in E corresponds to lipofuscin autofluorescence. Nuclear counterstaining was performed with 4′,6-diamidino-2-phenylindole (DAPI, blue).

**Figure 2.**
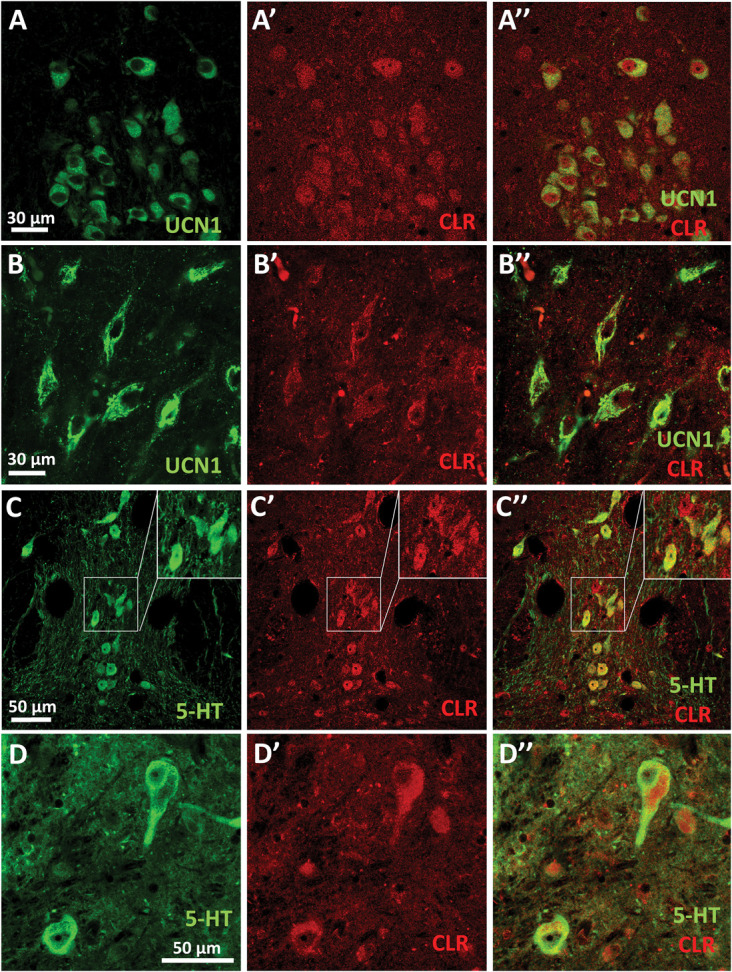
Calcitonin-like receptor (CLR) expression. (A–A″) Representative immunofluorescence images showing the CLR (red) coexpressed with urocortin 1 peptide (UCN1, green) in the mouse centrally projecting Edinger–Westphal (EWcp) nucleus. (B–B″) Representative immunofluorescence images showing the CLR (red) coexpressed with urocortin 1 peptide (UCN1, green) in the human EWcp. (C–C″) In the mouse dorsal raphe nucleus (DRN), the serotonin (5-HT, green) immunoreactive cells also contained CLR (red). (D–D″) In the human DRN, the 5-HT (green) immunoreactive cells coexpressed CLR (red).

**Figure 3.**
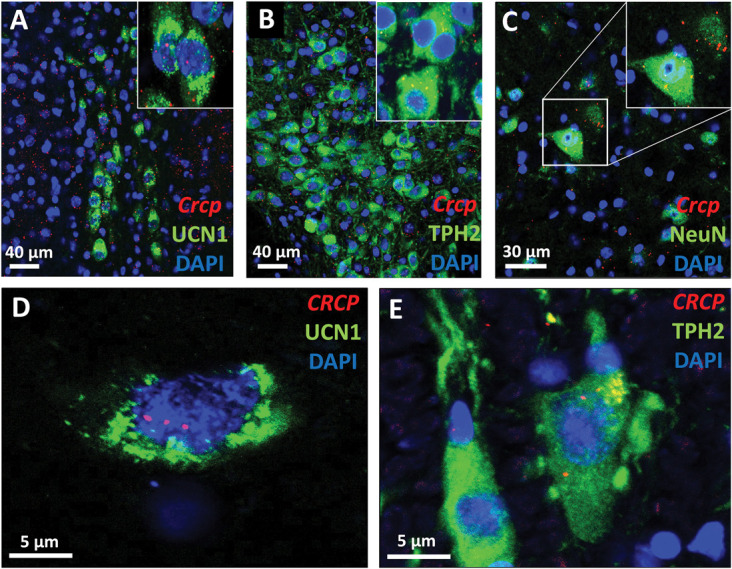
Calcitonin gene–related peptide receptor component (*Crcp*) mRNA expression. (A) Representative fluorescence images showing the *Crcp* mRNA transcripts (red) coexpressed with urocortin 1 peptide (UCN1, green) in the mouse centrally projecting Edinger–Westphal (EWcp) nucleus. (B) In the mouse dorsal raphe nucleus (DRN), the tryptophan hydroxylase-2 (TPH2, green) immunoreactive cells also contained *Crcp* mRNA transcripts (red). (C) Neurons (neuronal marker NeuN, green) of the mouse spinal trigeminal nucleus express also *Crcp* mRNA (red). (D) Representative fluorescence images showing the *CRCP* mRNA transcripts (red) coexpressed with UCN1 (green) in the human EWcp. (E) In the human DRN, the TPH2 (green) immunoreactive cells also contained *CRCP* mRNA transcripts (red). Nuclear counterstaining was performed with 4′,6-diamidino-2-phenylindole (DAPI, blue).

To confirm that elevated central CGRP levels may directly affect the urocortinergic EWcp neurons, we performed RNAscope ISH for *Calcr*, *Ramp1*, and *Crcp* mRNA moreover immunofluorescence for CLR and UCN1. We proved that *Ramp1* and *Calcr* mRNAs coding for AMY1 components are coexpressed both in mouse and in human EWcp neurons (Fig. 1). Notably, the expression pattern of *Calcr* mRNA in the EWcp suggests a substantial colocalization with UCN1 immunoreactive neurons. Moreover, almost all EWcp/UCN1 neurons were found to contain CLR (Fig. 2) and *Crcp* (Fig. 3) both in mice and humans.

In the DRN, RNAscope ISH in combination with immunofluorescence for *Calcr, Ramp1* (Fig. 1), and CLR (Fig. 2) as well as *Crcp* mRNAs (Fig. 3) were found to be expressed in TPH2-immunoreactive serotonergic and nonserotonergic neurons.

**Figure 4.**
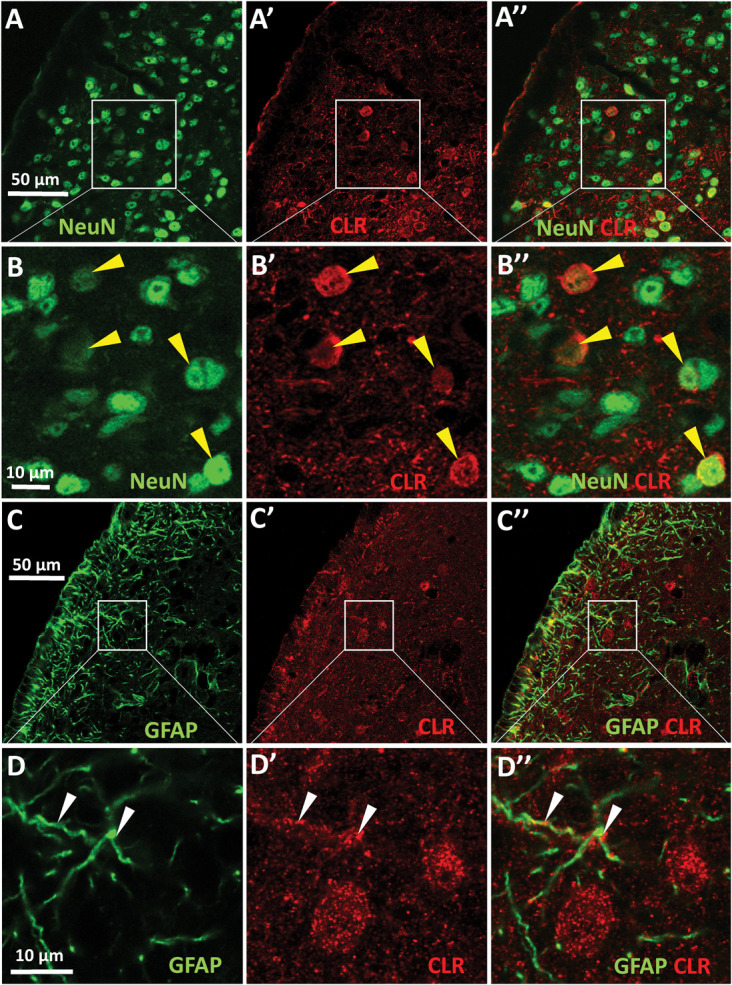
Calcitonin receptor-like receptor protein expression in mouse spinal trigeminal nucleus (STN). (A and B) The NeuN (green) labeling combined with CLR staining (red) revealed that a part of the STN neurons show also CLR immunoreactivity. Yellow arrowheads in the higher magnification images (B) depict the boxed areas in (A). (C and D) GFAP-CLR double labeling demonstrates that the GFAP (green)-immunoreactive astrocytes show also some CLR (red) immunopositivity. White arrowheads in the high magnification images in (D) demonstrating the marked areas in (C). The overlay images (A″–D″) in the right column demonstrate the colocalization of the labeled antigens. CLR, calcitonin-like receptor; GFAP, Glial fibrillary acidic protein.

We detected *Calcr, Ramp1* (Fig. 1), and *Crcp* mRNAs (Fig. 3) in both neuronal and glial cells in the mouse STN laminae I to III. In addition, in line with an earlier study, the CGRP receptor component CLR was confirmed in both neurons^71^ and astrocytes (Fig. 4).

#### 3.1.2. Urocortinergic afferentation of the *Crh1r-* and *Crh2r-*expressing spinal trigeminal nucleus neurons

**Figure 5.**
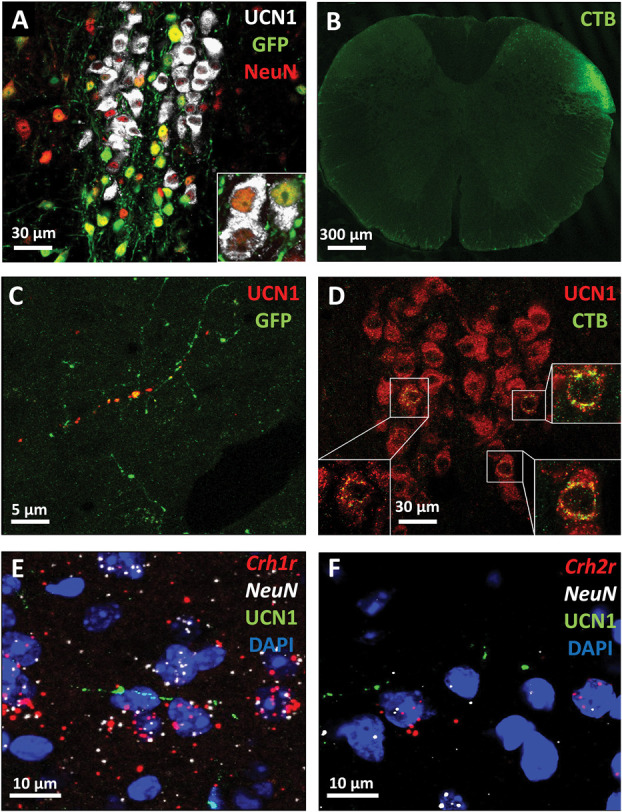
Urocortinergic afferentation from the centrally projecting Edinger–Westphal (EWcp) nucleus to the spinal trigeminal nucleus (STN). (A) Representative images showing the injection site of AAV8 Syn-enhanced green fluorescent protein (EGFP) by GFP (green) immunofluorescence in the EWcp urocortinergic (urocortin 1 [UCN1], white) neurons. All of the virus-infected cells are neurons (neuronal marker: NeuN, red), note the UCN1 and NeuN colocalization (yellow). (B) Fluorescence labeling for cholera toxin subunit B (CTB) in the dorsal horn of STN. (C) The colocalization of UCN1 (red) with GFP (green) in a nerve fiber in the STN and (D) the colocalization of UCN1 (red) with CTB (green) in the EWcp neurons. (E) Cells coexpressing neuronal marker mRNA (*NeuN*, white) and corticotropin-releasing hormone receptor 1 mRNA (*Crhr1*, red) as well as (F) corticotropin-releasing hormone receptor 2 (*Crhr2*, red) receiving a UCN1 (green)-positive afferentation in the STN. Nuclear counterstaining was performed with 4′,6-diamidino-2-phenylindole (DAPI, blue).

Anterograde and retrograde tracing studies were performed to investigate the possible urocortinergic projection from EWcp to STN. The accurate anatomical position of the injection site was approved by GFP and CTB immunostaining in EWcp (Fig. 5A) and C1 spinal cord sections (Fig. 5B), respectively. Green fluorescent protein and UCN1 double-positive fibers were observed in the STN in samples of mice subjected to AAV8-GFP injection into the EWcp (Fig. 5C). Cholera toxin subunit B and UCN1 double-positive neurons were detected in EWcp after a CTB injection into the STN (Fig. 5D).

Next, we examined whether neurons in the laminae I to III of STN express *Crhr1* and *Crhr2*, and they are approached by UCN1-immunoreactive nerve fibres. RNAscope ISH for *Crh1r, Crh2r*, and *NeuN* mRNA was combined with UCN1 immunofluorescence. Neurons in STN laminae I to III expressed both *Crhr1* (Fig. 5E) and *Crhr2* (Fig. 5F) mRNAs, and they were juxtaposed by urocortinergic fibres.

### 3.2. Model validation

#### 3.2.1. Light dark box test

**Figure 6.**
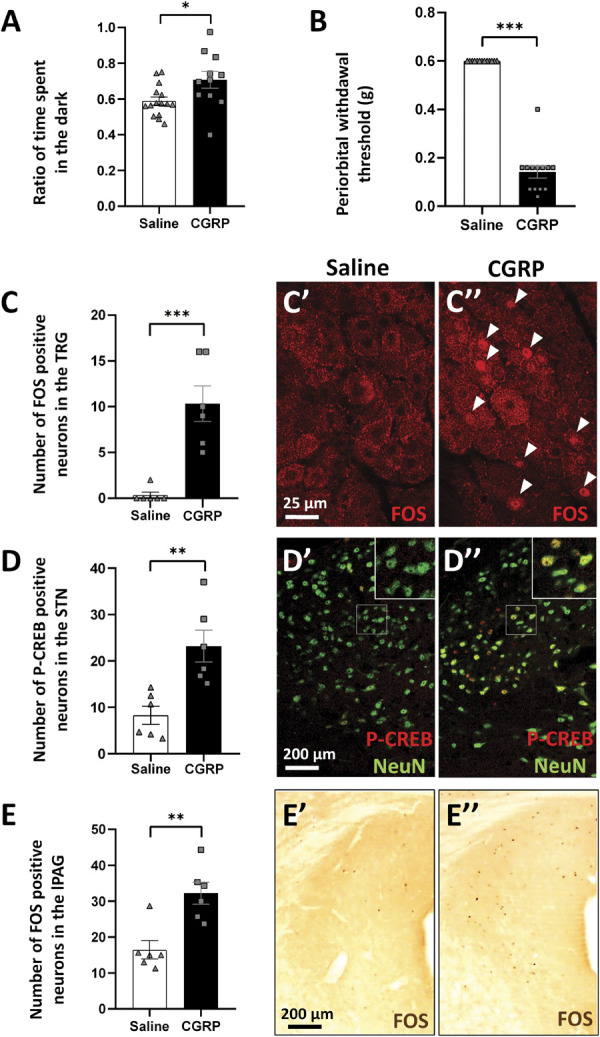
Model validation. (A) Columns show the ratio of time spent in the dark compartment of the light–dark box device, 30 minutes after saline (control) or calcitonin gene–related peptide (CGRP) injection (n = 11-15, *P = 0.02; Student *t* test). (B) Columns show the facial withdrawal threshold (g) in von Frey test, 30 minutes after saline (control) or CGRP injection (n = 13, ***P = 8.25531 × 10^−13^; Student *t* test). (C) Columns show the number of FOS positive neurons in the TRG (n = 6; ***P = 0.0004; Student *t* test). (C′ and C″) Representative fluorescence images showing the expression of FOS, as a marker of early neural activation in the trigeminal ganglion (TRG), 4 hours after saline (control) and CGRP injection. Nuclei of FOS-positive activated neurons (red) are highlighted by the white arrowheads. (D) Columns show the number of phosphorylated c-AMP–responsive element binding protein (P-CREB) positive neurons in the spinal trigeminal nucleus (STN) (n = 6; **P = 0.003; Student *t* test). (D′ and D″) Representative fluorescence images showing the expression of P-CREB, as a marker of neural activation in the STN, 4 hours after saline (control) and CGRP injection. Nuclei of P-CREB–positive activated neurons (red) are colocalized (yellow) with the neuronal marker (NeuN, green). (E) Columns show the number of FOS-positive neurons in the lateral periaqueductal gray matter (lPAG; n = 6; **P = 0.002; Student *t* test). (E′ and E″) Representative images showing the nuclei (brown dots) of activated FOS-positive neurons in the lPAG in control and CGRP-treated mice. FOS, c-fos gen–encoded protein; STN, spinal trigeminal nucleus.

Light–dark box test was performed to assess photophobia associated with migraine-like headache. Calcitonin gene–related peptide–treated mice spent significantly more time in the dark compared with the controls (Student *t* test, *P* = 0.02; Fig. 6A).

#### 3.2.2. Von Frey test

Von Frey test was performed to assess periorbital pain behavior associated with migraine-like headache. Calcitonin gene–related peptide treatment significantly reduced the periorbital withdrawal threshold compared with saline (Student *t* test, *P* = 8.25 × 10^−13^; Fig. 6B).

#### 3.2.3. Neuronal activation in the trigeminal ganglion, spinal trigeminal nucleus, and antinociceptive lateral periaqueductal gray matter

C-fos gene–encoded protein immunofluorescent labeling was performed to assess (1) the activation of the TRG neurons in the trigeminovascular nociceptive pathway and (2) the activation of the antinociceptive lPAG. To assess central sensitization and activation of STN neurons, immunofluorescence for FOS and P-CREB was performed.

Calcitonin gene–related peptide treatment resulted in a more than 10-fold increase in the number of FOS positive neurons in the TRG, compared with controls (Student *t* test, *P* = 0.0004) (Fig. 6C). In the STN, although CGRP treatment did not affect the FOS-positive neuron count (Student *t* test, *P* = 0.18), it increased the number of P-CREB (Student *t* test, *P* = 0.003) in laminae I to III (Fig. 6D). Calcitonin gene–related peptide treatment increased the FOS neuronal activation in the lPAG, compared with the control (Student *t* test, *P* = 0.002; Fig. 6E).

### 3.3. Activation of the centrally projecting Edinger–Westphal-circuit in the calcitonin gene–related peptide model

**Figure 7.**
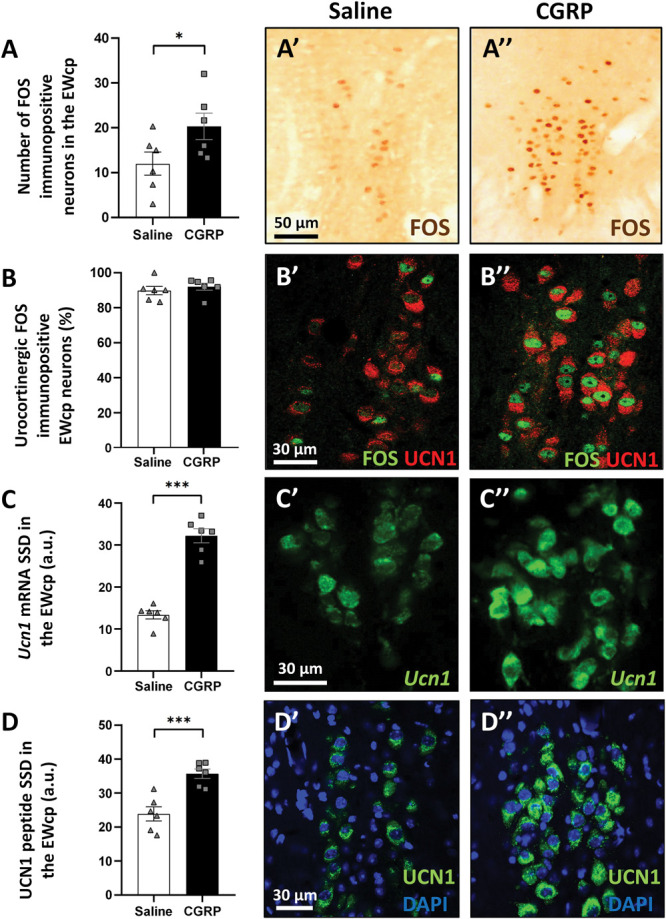
Response of the mouse centrally projecting Edinger–Westphal (EWcp) nucleus to calcitonin gene–related peptide (CGRP) treatment. (A) Columns show the number of FOS-immunoreactive neurons in the EWcp (n = 6; *P = 0.03; Student *t* test). (A′ and A″) Representative images showing the nuclei of the neurons positive (brown dots) for the early neural activation marker (FOS) in the EWcp, 4 hours after saline (control) or CGRP injection. (B) Quantitative evaluation of the ratio of urocortin 1 (UCN1)/FOS double-labelled neurons from all FOS-immunoreactive neurons in the EWcp revealed the urocortinergic identity of activated (FOS-immunoreactive) neurons. (B′ and B″) Representative fluorescence images showing the colocalization of UCN1 (red) and FOS (green) in the EWcp of control and CGRP-injected mice. (C) Quantitative evaluation of *Ucn1* mRNA-specific signal density (SSD) in the EWcp (n = 6; ***P = 0.00045; Student *t* test). (C′ and C″) Representative fluorescence images showing the expression of *Ucn1* mRNA (green) in the EWcp in control and CGRP-treated mice. (D) Quantitative evaluation of UCN1 peptide SSD in the EWcp (n = 6; ***P = 0.0007; Student *t* test). (D′ and D″) Representative images illustrate UCN1 peptide (green) immunoreactivity in the EWcp in control and CGRP-injected mice. For nuclear counterstaining, 4′,6-diamidino-2-phenylindole (DAPI, blue) was used. a.u., arbitrary unit; FOS, c-fos gene–encoded protein.

C-fos gene–encoded protein immunohistochemistry was performed to assess the neuronal activity of the EWcp. Calcitonin gene–related peptide treatment resulted in an approximately 2-fold rise in the number of FOS-positive neurons, compared with the control (Student *t* test, *P* = 0.03; Fig. 7A).

By a double-label immunofluorescence, we found that most of the FOS immunoreactive nuclei were localized to UCN1 neurons (91.79%), suggesting that the urocortinergic EWcp cells were activated (Fig. 7B).

Indeed, combined RNAscope ISH and immunofluorescence analysis confirmed that in CGRP-treated animals, both the *Ucn1* mRNA (Student *t* test, *P* = 0.0004) expression (Fig. 7C) and UCN1 peptide immunosignal (Student's *t* test, *P* = 0.0007; Fig. 7D) was significantly higher than in controls.

**Figure 8.**
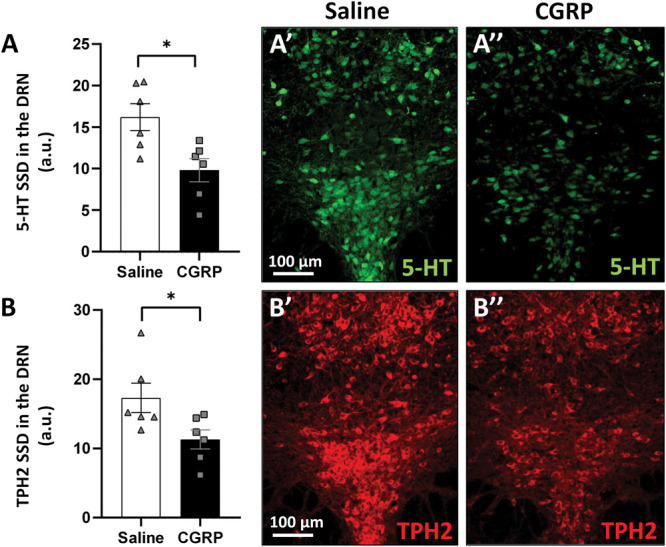
Serotonin (5-HT) and tryptophan hydroxylase-2 (TPH2) immunoreactivity in the dorsal raphe nucleus (DRN). (A) Specific signal density (SSD) of 5-HT in the DRN (n = 6; *P = 0.03; Student *t* test). (A′ and A″) Representative fluorescence images showing the 5-HT (green) immunoreactivity in the DRN, 4 hours after saline (control) and calcitonin gene–related peptide (CGRP) injection. (B) SSD of the TPH2 in the DRN (n = 6; *P = 0.01; Student *t* test). (B′ and B″) Representative fluorescence images illustrate TPH2 (red) immunosignal in the DRN. a.u., arbitrary unit.

In the DRN, CGRP treatment decreased both the 5-HT (Student *t* test, *P* = 0.03; Fig. 8A) and TPH2 (Student *t* test, *P* = 0.01; Fig. 8B) content of the serotonergic neurons.

### 3.4. Ablation of centrally projecting Edinger–Westphal/urocortin 1 neurons with leptin-conjugated saporin

**Figure 9.**
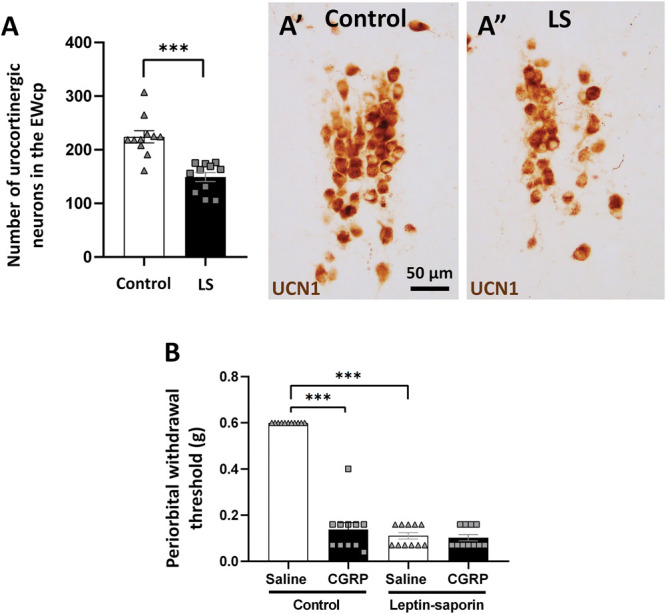
Ablation of urocortinergic (urocortin 1 [UCN1]) neurons in the centrally projecting Edinger–Westphal (EWcp) nucleus by leptin-saporin (LS). (A) Columns show the number of UCN1 neurons in the EWcp (n = 11; ***P = 0.0002; Student *t* test) in controls and LS-injected groups. (A′ and A″) Representative images showing the UCN1 neurons (brown) in the EWcp, in control and LS-injected mice. (B) Columns show the facial withdrawal threshold (g) in von Frey test, 30 minutes after saline or calcitonin gene–related peptide (CGRP) injection in control and LS-injected mice (n = 11, ***P = 0.0001; Tukey post hoc test upon repeated-measures ANOVA. ANOVA, analysis of variance.

Urocortin 1 immunohistochemistry was performed to confirm EWcp/UCN1 neuron loss. Leptin-conjugated saporin treatment significantly reduced the number of UCN1 immunoreactive neurons in the EWcp compared with the naive mice (*P* = 0.0002) (Fig. 9A). Von Frey test was performed to assess periorbital pain behavior associated with migraine-like headache. Before the ablation of EWcp/UCN1 neurons, CGRP treatment significantly reduced the periorbital withdrawal threshold compared with saline (*P* = 0.0001). Interestingly, after the ablation of EWcp/UCN1 neurons, the saline treatment significantly reduced periorbital withdrawal threshold compared with nonablated controls, (*P* = 0.0001), and CGRP treatment did not change it further (Fig. 9B).

### 3.5. Functional magnetic resonance imaging study

#### 3.5.1. Functional connectivity of Edinger–Westphal nucleus

**Figure 10.**
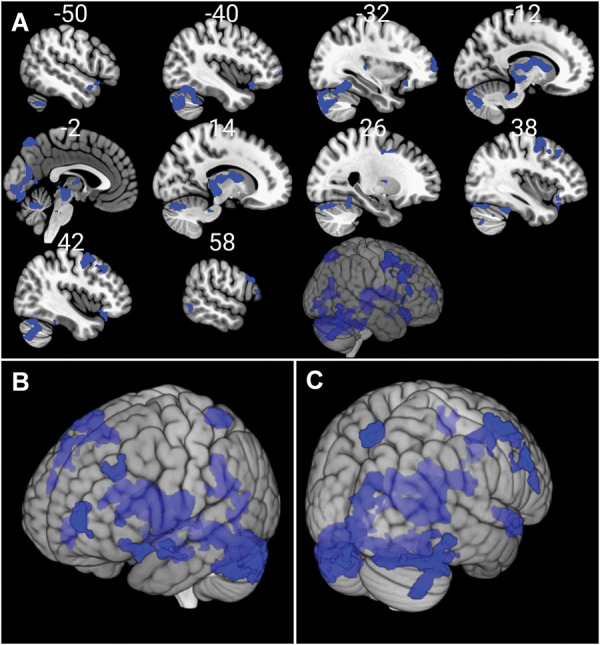
Functional connectivity matrix of Edinger–Westphal nucleus. Blue color represents functional connectivity of Edinger–Westphal nucleus with other brain areas: (A) sagittal reconstruction, lateral view; (B) left anterior aspect; (C) right posterior view. Significance threshold was cluster-level p(FWE) < 0.05 including at least 10 contiguous voxels. Results are corrected for age and sex. Statistical maps were visualized on the MNI 152 template brain provided in MRIcroGL (http://www.mccauslandcenter.sc.edu/mricrogl/). MNI, Montreal Neurological Institute.

Extensive positive functional connectivity of the EW nucleus with frontal and temporal gyri, cerebellum, caudate, and midbrain were identified in the whole population (Fig. 10 and Table 4). No significant negative connections were detected.

**Table 4:** Significant positive functional connectivity of Edinger–Westphal nucleus.

Cluster size (voxel)	Region	Peak coordinates			Peak
		x	y	z	T-value
5518	L thalamus	−2	−24	−8	17.99
	R lingual gyrus	12	−24	−6	5.882
	R caudate	14	−2	14	5.474
	L caudate	−10	10	6	4.91
	R thalamus	10	−20	10	4.69
	R pallidum	22	0	6	4.62
221	R precuneus	2	−72	56	5.410
319	L posterior cingulate cortex	−10	−34	−30	5.084
	R cerebellum III	12	−30	−30	4.672
1524	R cerebellum VI	36	−40	−28	5.027
	R cerebellum crus 2	42	−70	−44	4.660
278	R orbital part of inferior frontal gyrus	32	26	−14	4.973
596	R middle frontal gyrus	50	14	52	4.906
	R precentral gyrus	36	0	48	4.700
247	L superior temporal pole	−42	20	−16	4.905
173	L middle frontal gyrus	−24	28	34	4.534
196	R triangular part of inferior frontal gyrus	54	28	14	4.364
	R opercular part of inferior frontal gyrus	56	20	38	3.952
132	L middle temporal gyrus	−68	−30	−6	4.349
342	R medial part of superior frontal gyrus	2	56	34	4.331
	L medial part of superior frontal gyrus	0	52	46	3.898
230	L middle frontal gyrus	−30	60	8	4.159
	L orbital part of middle frontal gyrus	−38	56	−2	3.468

Reported results are significant at cluster-level p_FWE_ < 0.05. Coordinates are in Montreal Neurological Institute (MNI) space.

L, left hemisphere; R, right hemisphere.

Extensive positive functional connectivity of the EW nucleus with frontal and temporal gyri, cerebellum, caudate, and midbrain were identified in the whole population (Fig. 10 and Table 4). In accordance, significant positive functional connectivity was found between the EW and DRN (Peak pFWE = 0.004; Peak Z-value = 3.51, Peak MNI coordinates: x = 0, y = −28, z = −14) as well as STN (Peak pFWE = 0.007; Peak Z-value = 3.10, Peak MNI coordinates: x = 4, y = −40, z = −44) in whole group analysis. No significant negative connections were detected.

There was no significant difference between interictal migraine patients and healthy controls in the functional connectivity of EW nucleus after correction for multiple testing.

**Figure 11.**
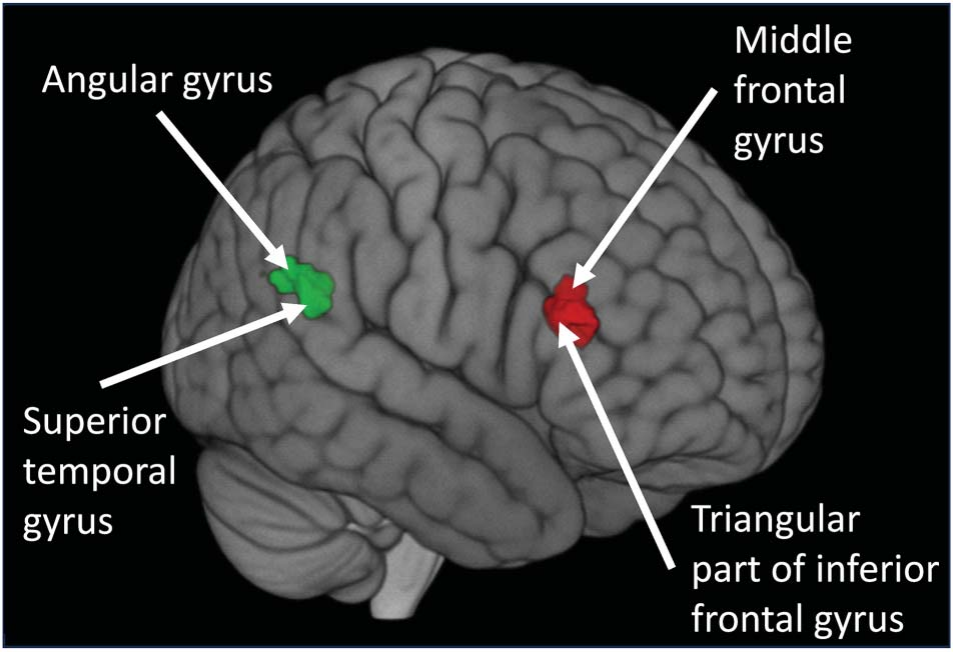
Brain clusters where functional connectivity of Edinger–Westphal nucleus positively correlated to migraine frequency. The green area represents the first cluster containing the angular gyrus and superior temporal gyrus, whereas red area is the other cluster containing the middle frontal and triangular part of inferior frontal gyri. Significance threshold was cluster-level p(FWE) < 0.05 including at least 10 contiguous voxels. Results are corrected for age and sex. Statistical maps were visualized on the MNI 152 template brain provided in MRIcroGL (http://www.mccauslandcenter.sc.edu/mricrogl/). MNI, Montreal Neurological Institute.

However, positive correlation was found between migraine frequency and functional connectivity of EW nucleus with 2 clusters. One cluster (cluster size = 123 voxel, pFWE = 0.045) contained the angular gyrus (Peak T-value = 4.211, Peak MNI coordinates: x = 54, y = −52, z = 26) and superior temporal gyrus (Peak T-value = 3.961, Peak MNI coordinates: x = 58, y = −40, z = 22), whereas the other cluster (cluster size = 166 voxel, pFWE = 0.011) contained the middle frontal (Peak T-value = 3.994, Peak MNI coordinates: x = 54, y = 34, z = 22) and triangular part of inferior frontal gyri (Peak T-value = 4.189, Peak MNI coordinates: x = 48, y = 30, z = 14; Fig. 11).

## 4. Discussion

The main goal of this study was to confirm the involvement of EWcp in migraine. The central distribution of CGRP and that of its receptor has been described at protein level in rats, mice, and humans.^27,43,64^ However, CGRP receptors AMY1 and CGRP receptor components expression in EWcp has not been studied yet. Here, we have confirmed the expression of *Ramp1*, *Calcr*, and *Crcp* mRNA as well as CLR peptide in EWcp/UCN1 neurons using the highly sensitive and specific RNAscope ISH technique combined with immunofluorescence^101^ in both mice and humans. This supports the translational relevance of our animal work and confirms findings of earlier mRNA studies.^27,64^ Because AMY1 and CGRP receptor components were also detected in the migraine-related EWcp projection areas such as DRN and STN, we assume that the CGRP may activate the DRN and STN directly or indirectly through AMY1 and CGRP receptor-expressing EWcp/UCN1 neurons. Indeed, both our anterograde and retrograde tracing proved the direct projection from the EWcp to the STN. Because urocortinergic fibers were detected in the close proximity of STN neurons, which expressed *Crh1r* and *Crh2r* mRNAs, we provide neuroanatomical evidence for a direct urocortinergic EWcp projection to STN neurons with a possible functional significance.

Next, we investigated the response of EWcp in a migraine model. Several well-validated in vivo models were described, including direct electrical stimulation of trigeminal neurons, administration of inflammatory substances to the meninges, and the use of algogenic substances (eg, nitroglycerin, CGRP).^41^ Importantly, the CGRP signaling is involved in all these models.^104^ Electrical stimulation of TRG and meningeal administration of inflammatory substances are invasive, and considering the acute pain sensitivity of EWcp,^56,84^ these models were not suitable. Intraperitoneal administration of nitroglycerin and CGRP successfully resembled many aspects of acute and chronic migraine^41,53^ in rodents that were alleviated by antimigraine drugs like sumatriptan.^9,30,81,104^ In our project, the use of nitroglycerin would have been unreliable because all available preparations contain ethanol as vehicle,^9,30,53^ and the EWcp is highly alcohol sensitive.^1,109^ Therefore, we decided to use the intraperitoneal CGRP treatment model of migraine described by Mason et al.^68^ Several migraine-related symptoms have been observed upon central and peripheral CGRP administration in humans and rodents,^52,60,68^ suggesting both peripheral and central site of action; this is in line with the wide distribution of CGRP and AMY1 receptors in the CNS and periphery.^27,45^ TRG neurons express CGRP and its receptor, and TRG provide anatomical connection between peripheral and CNS. Activation of CGRP receptors in trigeminal neurons stimulate CGRP release from (1) peripheral nerve endings of afferent terminals, which innervate meninges, (2) TRG cell bodies, and (3) terminals of central processes in the STN.^25^ Calcitonin gene–related peptide release from central terminals promote central sensitization through CGRP receptors on second-order nociceptive STN cells,^25,46^ which may ultimately lead to further central CGRP release from CGRP-expressing brain areas. This is one mechanism for central sensitization mediated by peripheral action of CGRP, but several others may also contribute.^45,46,68^

The CGRP model^68^ was validated by the periorbital hyperalgesia and light aversion behavior. According to Mason et al., calcitonin gene–related peptide treatment did not induce anxiety in mice; thus, the LDB behaviour can be considered as photophobia, referring to a migraine-like state. Furthermore, CGRP-induced light aversion did not correlate with the blood pressure excluding vasomotor mechanisms.^69^

The elevated TRG/FOS and STN/P-CREB immunosignals upon CGRP treatment refers to the activation of these key players in migraine pathogenesis.^29,46^ Increased lPAG/FOS immunosignal in CGRP-treated mice suggests that the activation of the descending antinociceptive pathways further reinforce that a migraine-like headache has occurred.

The neuromodulatory role of PAG in migraine is well known,^73,99^ but no studies have reported the contribution of EWcp to migraine, although it is located within the PAG. The two-fold increase in the number of FOS-positive EWcp/UCN1 neurons upon CGRP treatment suggests increased neuronal activation that requires adaptation at the level of gene expression.^1,36^ This was concomitant with an increased *Ucn1* mRNA and UCN1 peptide content in response to CGRP administration, suggesting higher UCN1 peptide release. We propose that peripherally administered CGRP may activate the EWcp/UCN1 neurons directly by centrally released CGRP or by indirect manner as a consequence of central sensitization. The presumably increased UCN1 release may directly influence the function of the STN through CRH1R and CRH2Rs, as supported by increased P-CREB immunosignal in the STN. In line with earlier studies,^11,12^ no FOS activation was observed in the STN upon CGRP treatment.

Beyond the STN, the DRN is also a brain area known to be activated in migraine.^88^ The EWcp/UCN1 neurons innervate the DRN,^23,97,109^ where both CRH receptors are expressed.^14,64,96,97^ Activation of CRH1R in DRN reduces 5-HT release, whereas CRH2R signaling has opposite effect.^33,63^ The decreased DRN/5-HT content in our CGRP-treated mice suggests that 5-HT release upon CGRP administration. Supporting this, elevated CNS 5-HT levels were found during migraine attacks.^17,24,80,89^ We propose that CGRP increased the UCN1 release in the DRN that through CRH2Rs of DRN/5-HT neurons ultimately elevated 5-HT release. Nevertheless, we cannot exclude a direct CGRP effect on serotonergic neurons as they also express CGRP receptors.^64^

Calcitonin gene–related peptide treatment decreased the TPH2 enzyme level in DRN. Tryptophan hydroxylase-2 is the rate-limiting enzyme of central 5-HT synthesis,^100^ and its gene polymorphisms have been linked to migraine.^51,67^ Chronic central UCN1 microinjection elevated the *Tph2* mRNA expression in the caudal and dorsal subdivision of the DRN, whereas an opposite effect was observed in the ventrolateral part.^22^ This finding is in contrast with our results as we saw a rise in UCN1 levels but decreased the TPH2 protein content upon CGRP treatment. This discrepancy may be explained by methodological differences as in our study, we examined the TPH2 at protein level. Conversely, we applied a single CGRP treatment, which elevated the UCN1 level, whereas Donner et al.^22^ applied chronic UCN1 administration. Thirdly, a DRN subdivision-specific difference in CRH2R expression, moreover a stress exposure-dependent change in the CRH1R and CRH2R receptor trafficking and consequent ligand availability in the plasma membrane.^103^ may explain the difference in the outcome of these studies. All in all, the complex action of UCN1 on DRN serotonergic neurons may reflect the proclaimed abnormalities in central 5-HT turnover in migraine.^24,80^

Given that the cocaine and amphetamine-regulated transcript and EWcp/UCN1 show full colocalization,^78,109^ decreased periorbital withdrawal threshold following EWcp/UCN1 ablation aligns with earlier study result where increased the activity of EWcp/cocaine and amphetamine-regulated transcript resulted in increased paw withdrawal threshold, suggesting that these neurons modulates pain.^78^

The ability of EWcp to influence migraine-related areas through direct neuroanatomical connection was further supported by the significant positive functional connectivity between EW, DRN, and STN. To the best of our knowledge, this is the first study to describe the functional connectivity of EW in the human brain. Moreover, our fMRI study revealed a strong positive correlation between the frequency of migraine attacks and the functional connectivity of EW with brain areas that are known to be part of the affective pain pathway including angular gyrus, superior temporal gyrus, middle frontal and the triangular part of inferior frontal gyri,^48,76,102^ supporting previous findings that changes in these brain areas may predispose a person to pain conditions including migraine.^48^

As to the limitations, none of the models for migraine can reliably mimic all aspects of human disease and the CGRP model is also not an exception. Although migraine is more prevalent in females,^83^ we used male mice because (1) EWcp/UCN1 neurons express estrogen receptor beta and estrous cycle-related hormonal fluctuations modulate UCN1 levels^18,19^ and (2) 0.1 mg/kg CGRP induced migraine-like symptoms in both male and female mice.^68,81^ Our fMRI study recruited male and female migraineurs only during the interictal period; thus, the results do not reflect the brain status during the active headache episodes. This may explain the lack of significant difference in the functional connectivity of EW between the study groups. Edinger–Westphal seed has not been described earlier.^89^ In this study EW seed definition was carried out based on recent literature data^108^ and confirmed by the visual check of expert scientists.

With respect to the limitations above, we conclude that the EWcp was activated in the CGRP model of migraine associated with increased *Ucn1* mRNA expression and UCN1 peptide content in male mice. We found a direct EWcp-derived urocortinergic input to the STN and demonstrated that the STN and DRN neurons express *Crh1r* and *Crh2r* mRNAs. Hence, we propose that elevated central CGRP and increased UCN1 release from EWcp/UCN1 neurons in response to CGRP may directly modulate the function of STN and DRN. This suggests the role of EWcp in the endogenous response to migraine. In line with these, we have also revealed a significant positive functional connectivity between EW and STN as well as DRN by fMRI in humans. In our ongoing research, we are investigating the EWcp/UCN1 in antimigraine therapy too.

## Conflict of interest statement

The authors declare that the research was conducted in the absence of any commercial or financial relationships that could be construed as a potential conflicts of interest.

The datasets used and/or analyzed during the current study are available from the corresponding author on reasonable request.

All authors read and approved the final version of the manuscript.

## Appendix A. Supplemental digital content

Supplemental digital content associated with this article can be found online at http://links.lww.com/PAIN/C72.

## Supplementary Material
