# Supplementary material for: Neuroanatomical evidence and a mouse calcitonin gene–related peptide model in line with human functional magnetic resonance imaging data support the involvement of peptidergic Edinger–Westphal nucleus in migraine
Source: Pain. 2024 Jun 14;165(12):2774–93. doi: 10.1097/j.pain.0000000000003294 (PMC11562765; doi:10.1097/j.pain.0000000000003294)
Supplement: Supplementary file 1 [file jop-165-2774-s001.pdf]

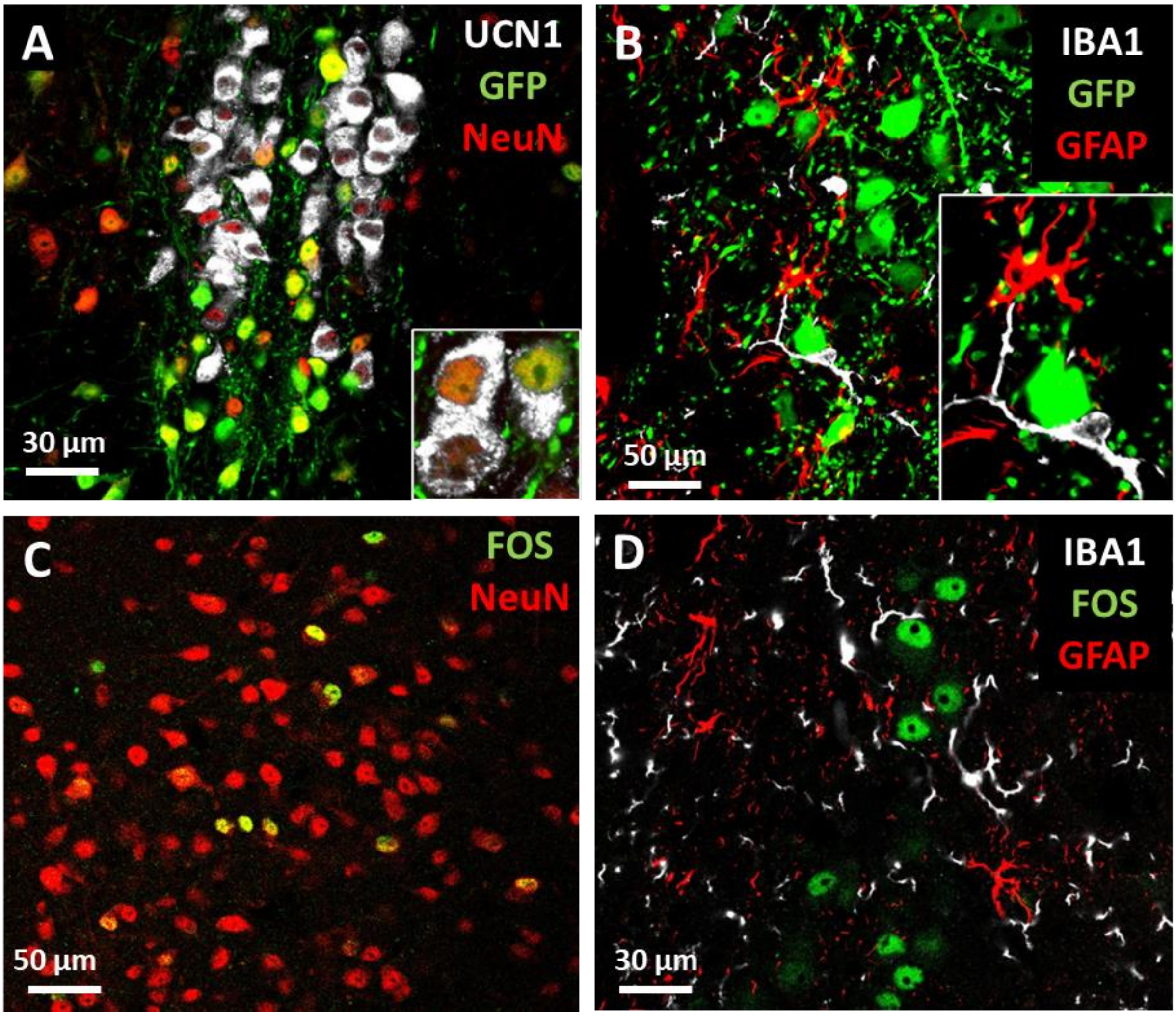

Supplementary figure 1. Histological characterization of adeno-associated virus (AAV)-infected cells in the centrally projecting Edinger-Westphal nucleus (EWcp) and FOS immunoreactive cells in the mesencephalic lateral periaqueductal gray matter (lPAG). (A) AAV-infected cells in the EWcp were visualized by immunofluorescence for green fluorescence protein (GFP, green in A and B). Note that the green GFP immunopositive cells contain also the neuronal nuclear marker (NeuN, red) as shown also by the higher magnification insert in A. Most of the GFP and NeuN immunopositive cells co-express urocortin1 (UCN1, white in A) also, proving the neuronal identity of AAV-infected peptidergic cells in the EWcp. (B) The AAV-infected, GFP-immunopositive cell bodies in the EWcp do not correspond to glial cells as they contain neither the microglial marker ionized calcium-binding adapter molecule 1 (IBA1, white in B) nor the astroglial marker, glial fibrillary acidic protein (GFAP, red in B). (C) Immunohistological confirmation of neuronal identity of FOS-immunoreactive cells (green in C and D) in the periaqueductal gray matter upon CGRP

treatment as the FOS signal fully co-localizes with the neuronal marker NeuN (red in C). (D) The nuclear FOS (green) signal is not localized to micro- (IBA1, white) or astroglial (GFAP, red) cells.

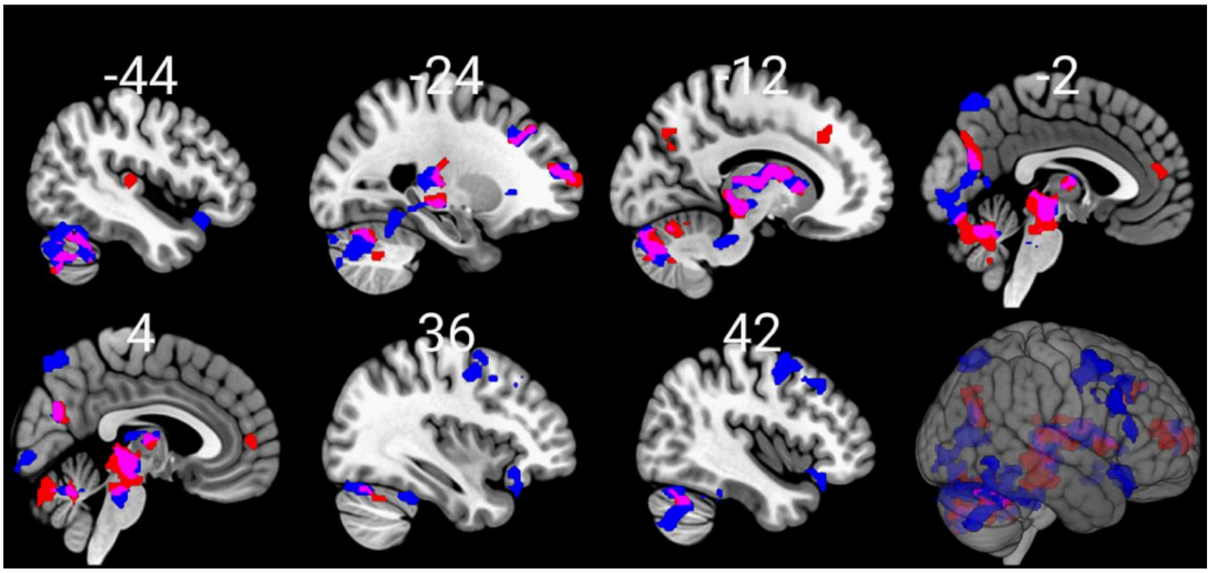

Supplementary figure 2. Similarities and differences in functional connectivity of Edinger-Westphal nucleus and periaqueductal gray matter. Blue color represents functional connectivity of Edinger-Westphal nucleus; red color represents functional connectivity of periaqueductal gray matter; pink color represents overlapping connectivity. Initial threshold of $p < 0.001$ uncorrected for multiple comparison and at least twenty contiguous voxels was used in the analysis. All connections were positive.

**Case code**	**Gender**	**Age**	***Post mortem* time (min)**
SKO27	Female	76 Years	195
SKO28	Female	93 Years	224
SKO29	Female	69 Years	212

Supplementary table 1. Characteristics of human brain samples used for RNAscope *in situ* hybridization.

Cluster size (voxel)	Region	Peak coordinates			Peak
		x	y	z	T-value
2855	Midbrain	4	-28	-6	16.100
	Midbrain	18	-22	-8	5.677
	Midbrain	-24	-22	-6	4.810
222	L Superior frontal gyrus	-26	62	10	5.328
	L Middle frontal gyrus	-24	50	16	4.097
1468	R Vermis_7	2	-74	-26	4.66
	R Cerebelum_IX	12	-54	-34	4.62
	L Cerebelum_VI	-26	-60	-36	4.499
171	L Cerebelum_Crus1	-40	-46	-36	4.506
170	R Anterior cingulate cortex	12	50	8	4.318
	L Anterior cingulate cortex	-2	48	12	3.593
318	L Calcarine	-2	-68	22	4.313
	L Precuneus	-2	-72	32	3.849
	R Precuneus	8	-66	30	3.601
173	L Superior frontal gyrus	-12	28	36	4.111
	L Middle frontal gyrus	-24	36	40	3.659
192	L Cerebelum_Crus1	-46	-58	-38	4.111
	L Cerebelum_Crus2	-40	-74	-46	4.015

Supplementary table 2. Regions with positive functional connectivity of periaqueductal gray matter. Reported results are significant at cluster-level $p_{FWE} < 0.05$. Coordinates are in Montreal Neurological Institute (MNI) space. R: right hemisphere, L: left hemisphere.
